# Role of electronic health literacy and insight into cancer among health workers and patients: a review

**DOI:** 10.3389/fpubh.2026.1781759

**Published:** 2026-05-29

**Authors:** Yuehan Zhang, Zijing Wu

**Affiliations:** China Medical University, Shenyang, China

**Keywords:** cancer care, digital health literacy, eHealth literacy, information appraisal, oncology, patient engagement, scoping review

## Abstract

**Background:**

Digital technologies in oncology require patients and health workers to possess adequate electronic health literacy (eHealth literacy). Yet, the extent to which this literacy influences cancer-specific insights, ranging from knowledge to clinical behavior, remains fragmented.

**Objective:**

This scoping review systematically mapped the literature on eHealth literacy across oncology populations and characterized its associations with cognitive, behavioral, evaluative, and psychological outcomes.

**Methods:**

Adhering to PRISMA-ScR, we searched PubMed and Scopus through December 15, 2025. We included English-language primary studies measuring eHealth literacy in oncology populations and linking it to outcomes like screening or adherence.

**Results:**

Among 65 included studies, 50% focused on patients, with limited attention to survivors (9.7%) or the workforce (6.5%). The eHealth Literacy Scale (eHEALS) was the dominant metric (66%), while performance-based instruments were rare. Evidence clustered heavily around proximal outcomes such as digital readiness and information appraisal. Conversely, distal endpoints, including adherence and quality of life, were underrepresented. While associations with information engagement were positive, evidence linking literacy to clinical behaviors remains sparse.

**Conclusion:**

eHealth literacy is a key determinant of digital engagement; however, the evidence base is limited by the predominance of cross-sectional designs, reliance on self-reported measures, and substantial heterogeneity across studies. Advancing the field requires longitudinal designs, objective assessments, and a focus on the oncology workforce.

## 1 Introduction

### 1.1 Digital information environments in contemporary oncology care

Cancer care increasingly unfolds in a dense information environment where patients, survivors, caregivers, and clinicians must make decisions under uncertainty, time pressure, and emotional strain (1–3). Beyond understanding a diagnosis, people often need to compare treatment options, interpret complex test results such as genetic or immunological markers, anticipate side effects, and decide when to seek help for disease complications or emerging risks (3–8). In parallel, many patients turn to the internet to reduce uncertainty, seek reassurance, and gain a sense of control, and their information needs can shift across the disease trajectory from primarily cognitive questions to more affective concerns (9, 10).

Digital services now sit inside routine care workflows. In oncology, portal timing and content can shape what patients learn first, how quickly they encounter new information (sometimes before a clinical explanation), how they prepare for visits or consultations, and how they coordinate care across multiple services (10, 11).

At the same time, digital tools are increasingly used to support shared decision making, including structured decision aids, communication tools, and training interventions. Meanwhile, newer AI-enabled decision aids are being explored to personalize risk and option information; users often report benefits such as clarity and ownership, while clinicians raise concerns about bias, integration, and whether recommendations stay current. Together, these trends make the ability to navigate, evaluate, and apply digital cancer information a practical determinant of patient understanding, engagement, and downstream outcomes that oncology care aims to improve (12–13).

### 1.2 Key definitions and analytic framing

Clear terminology is essential because “digital engagement” can mean anything from passive browsing to skilled application of complex health information. In this review, we use eHealth literacy to mean the skills needed to locate digital health information, interpret it, judge its credibility and relevance, and use it to guide health decisions and actions (14, 15). In this article, we also use the term cancer survivor to refer to any individual from the time of a cancer diagnosis onward, regardless of treatment phase or prognosis (16). The term “cancer insight” is not used here as a psychiatric construct. Instead, we operationalize it as measurable outcomes that reflect understanding, appraisal, and effective use of cancer-related information. Consistent with our extraction framework, “insight” outcomes are grouped into: (1) cognitive outcomes (cancer knowledge, symptom awareness, risk or treatment understanding), (2) behavioral outcomes (screening participation, treatment adherence or self-management behaviors), (3) evaluative outcomes (ability to judge information quality, including misinformation identification), and (4) psychological outcomes that shape decision-making capacity (such as anxiety, empowerment, or self-efficacy). This framing preserves conceptual clarity while allowing heterogeneous primary studies to be mapped into comparable outcome domains.

### 1.3 Rationale and gap

Although digital access is now woven into oncology care, the ability to benefit from that access is uneven. Cancer information environments are increasingly shaped by social media and other “new media” channels where the quality of cancer-related content varies widely and misinformation can be prominent, creating risks for vulnerable patients who are making high-stakes decisions (17, 18). At the same time, clinical systems are shifting information disclosure toward patients through portals and immediate release of results, yet oncology portal access and use show measurable disparities by sociodemographic and clinical factors, suggesting that digital access does not automatically translate into informed engagement (19).

Within this context, eHealth literacy and related digital health literacy constructs are frequently proposed as modifiable determinants of how people interpret, evaluate, and act on online cancer information. However, the existing evidence base is fragmented in ways that limit actionable conclusions.

The objectives of this scoping review were to: (1) map how eHealth literacy has been measured in oncology populations; (2) categorize the types of outcomes linked to eHealth literacy; and (3) identify gaps in populations, measurement approaches, and outcome domains to inform future research and practice.

## 2 Methods

### 2.1 Design and reporting standards

We selected a scoping review approach to describe and organize the available evidence linking eHealth literacy to cancer-relevant outcomes, with an emphasis on what has been studied, how it has been measured, and where evidence is missing, rather than on computing a single pooled effect estimate. This review was not prospectively registered. Reporting was guided by the PRISMA extension for scoping reviews (PRISMA-ScR) to ensure complete and reproducible documentation of the search, selection process, data charting, and synthesis (20). Where relevant, we also aligned methodological decisions with contemporary scoping review guidance from the JBI Manual for Evidence Synthesis, particularly for describing eligibility criteria, selection processes, and evidence mapping outputs (21).

### 2.2 Eligibility criteria

Eligibility criteria were developed *a priori* using a Population-Concept-Context (PCC) framework, consistent with established guidance for scoping reviews and PRISMA-ScR reporting (20). We included studies focused on (i) cancer patients (any cancer type, stage, or treatment phase), (ii) cancer survivors, and (iii) health workers involved in oncology care (for example, oncology nurses, oncologists, or allied professionals working with cancer patients). In addition, community-based samples were eligible only when the target of investigation was explicitly cancer screening or cancer awareness (not general health), and when the cancer context was central to the research question (for example, screening participation or intention for a specified cancer).

Studies were eligible only if they explicitly measured eHealth literacy or digital health literacy, defined as skills and competencies related to locating, understanding, evaluating, and applying digital health information or services. Acceptable operationalizations included validated instruments (for example, eHEALS, DHLI, eHLQ-related tools) or clearly described, construct-consistent measures. To meet inclusion, studies had to examine the relationship between eHealth literacy (or digital health literacy) and at least one cancer-relevant outcome domain aligned with our analytic framing, including: cancer knowledge or awareness, symptom awareness, screening participation or intention, treatment adherence or self-management, ability to evaluate information quality or identify misinformation, decision-making outcomes (for example, decisional conflict or shared decision-making measures), or psychological outcomes relevant to insight and engagement (for example, anxiety, empowerment, self-efficacy). Studies that measured eHealth literacy but did not link it to any outcome domain (for example, purely descriptive assessments without outcome association) were excluded.

We included studies from any country and setting, including clinical (inpatient, outpatient, specialty oncology centers), community-based screening contexts, and online environments, reflecting the goal of mapping evidence across diverse care and information systems.

Studies were included if available in English and provided sufficient detail for data charting. No publication date restrictions were applied.

### 2.3 Information sources and search strategy

We searched PubMed (MEDLINE) and Scopus from their earliest indexed records through 15 December 2025. These databases were selected due to their broad and complementary coverage of biomedical, public health, and interdisciplinary research, including oncology and digital health domains. The search strategy combined controlled vocabulary and free-text terms related to eHealth literacy (e.g., “eHealth literacy,” “digital health literacy,” “eHEALS,” “DHLI,” “eHLQ”) and oncology (e.g., “cancer,” “neoplasm,” “oncology,” “tumor”). The full search strategy is provided in Supplementary Appendix S1. All retrieved records were exported, merged, and deduplicated prior to screening.

### 2.4 Selection process

All records retrieved from PubMed and Scopus were exported on the day of the final search and combined in a single master library. Duplicate citations were removed prior to screening using a structured approach based on unique identifiers (DOI, PMID where available) and normalized title matching. The deduplicated set was then screened using the predefined eligibility criteria (Table 1).

**Table 1:** Cross-tabulation of study populations by cancer type.

Population group	Mixed/any cancer	Breast	Colorectal	Lung	Prostate	Cervical	Skin/melanoma	General cancer survivorship	No specific site focus	Online cancer information	Smoking cessation, sun safety, diet/PA, screening	Cancer health literacy, not site-specific	Outcome is use of digital technologies for cancer prevention	Attitudes toward general cancer screening	Rheumatoid arthritis, non-cancer population	Rheumatoid arthritis cohort	COVID-19 general population	General health topics	General population; cancer history used as covariate	non-muscle invasive bladder cancer	Total (studies)
Patients	11	4	0	1	2	3	1	0	1	0	0	1	1	1	1	1	0	0	0	1	29
Survivors	5	1	0	1	1	0	0	1	0	0	0	0	0	0	0	0	0	0	0	0	9
Oncology health workers	2	1	0	0	0	0	0	0	0	0	0	0	0	0	0	0	0	0	0	0	3
Screening population	0	0	0	0	0	1	0	0	0	0	0	0	0	0	0	0	0	0	0	0	1
Mixed/other	1	1	2	0	0	2	1	0	0	1	1	0	0	0	0	0	1	1	1	0	12
Total (studies)	19	7	2	2	3	6	2	1	1	1	1	1	1	1	1	1	1	1	1	1	54

Title/abstract and full-text screening were conducted independently by two reviewers using the predefined eligibility criteria. Discrepancies were resolved through discussion, and where necessary, consultation with a third reviewer.

Full texts were retrieved for all records passing stage one and for those where abstracts did not provide sufficient information to determine eligibility.

**Figure 1:**
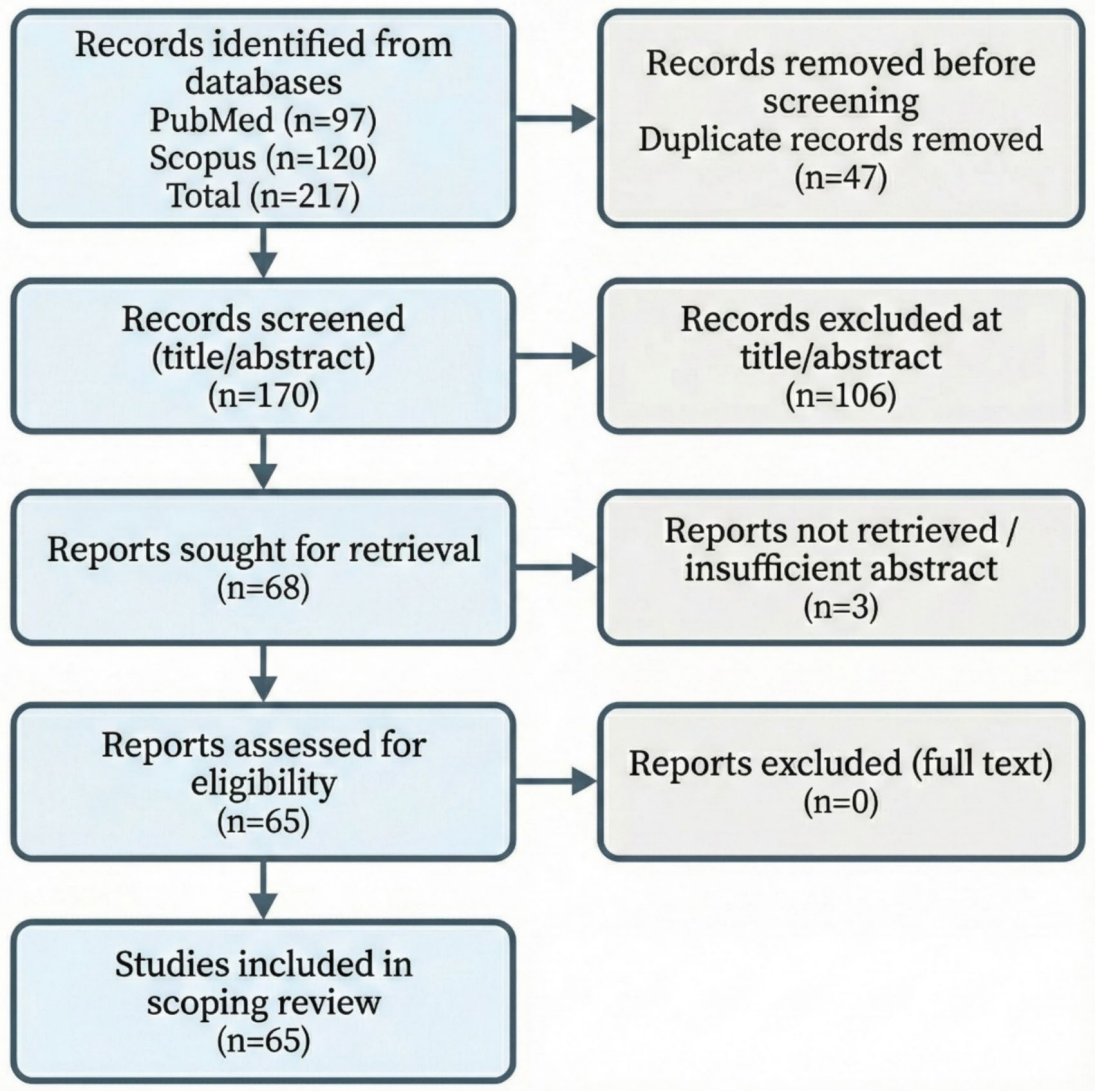
PRISMA flow diagram detailing the study selection process.

The numbers of records at each step (identified, deduplicated, screened, assessed in full text, and included) are presented in Figure 1, consistent with PRISMA-ScR guidance (20).

### 2.5 Data charting and extraction process

In line with scoping review conventions, data extraction was conducted as data charting, using a structured form designed to capture variables required to address the review questions and to support transparent evidence mapping. The charting form was piloted on a subset of included studies and refined to ensure consistent handling of common patterns (multiple outcomes, multiple tools, or multiple cancer groups).

**Figure 2:**
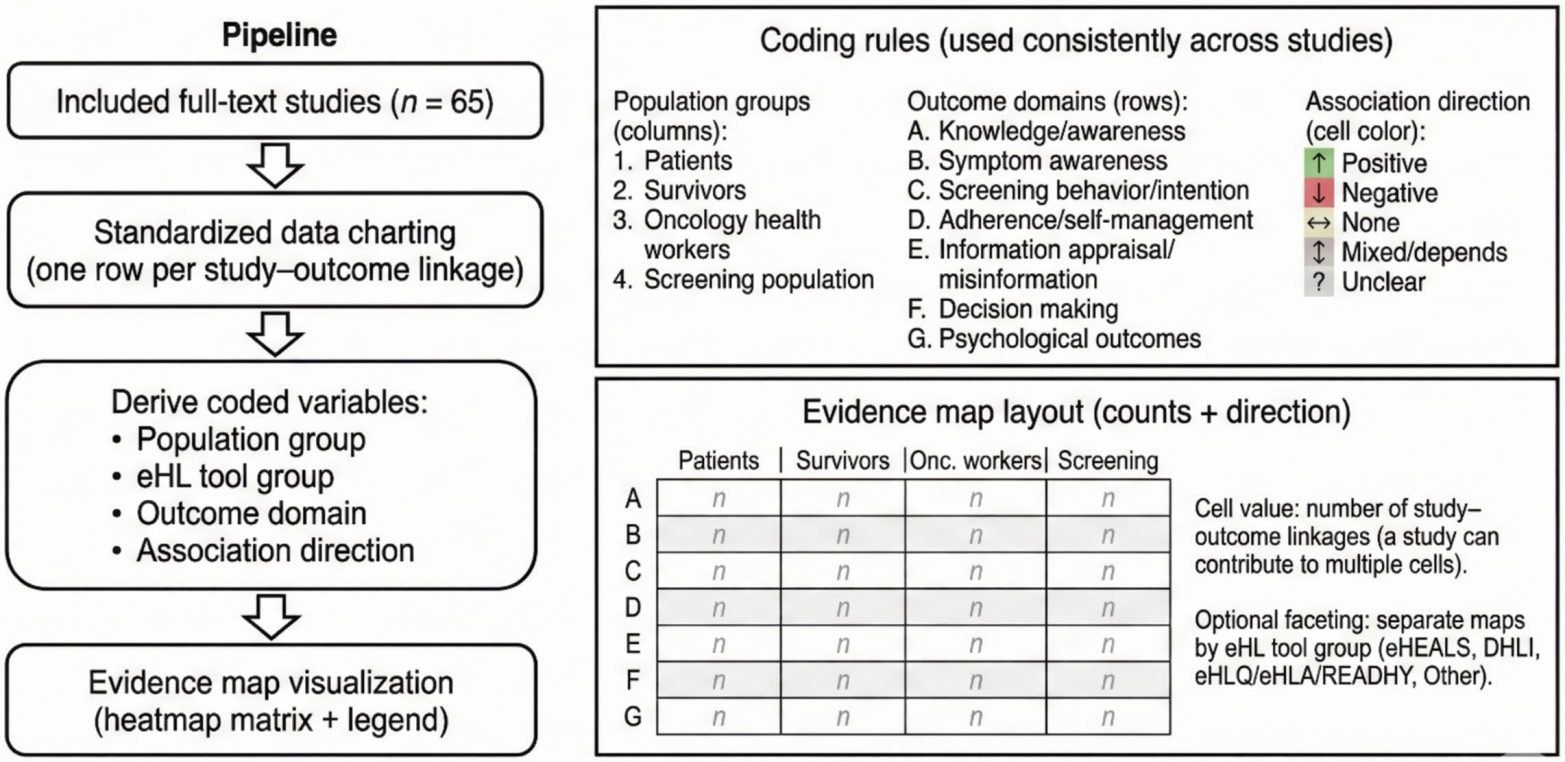
Design schema and coding rules used for the evidence map.

Before full charting, the form was piloted on a small sample of included studies and refined to improve clarity of field definitions, reduce ambiguity, and ensure consistent handling of common reporting patterns (Figure 2).

### 2.6 Synthesis approach and evidence mapping

**Figure 3:**
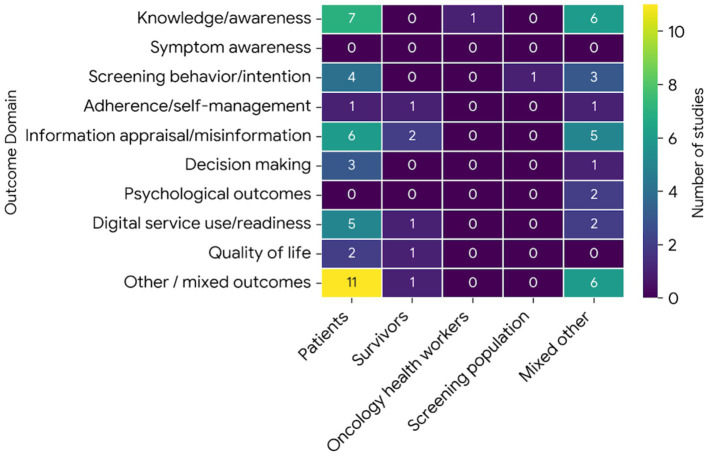
The distribution of evidence across population groups and outcome domains, with color coding indicating the direction of association. Higher-density cells reflect areas with greater research concentration, while sparse cells highlight gaps in the literature.

We synthesized findings using descriptive evidence mapping and narrative integration, consistent with scoping review methodology; therefore, no meta-analysis was conducted. We summarized study characteristics using frequencies and cross-tabulations (year, country/region, design, population type, cancer context, and measurement tools). Outcomes were coded into predefined domains aligned with our analytic framing, and each distinct eHealth literacy-to-outcome linkage was retained for mapping when studies reported multiple outcomes. For quantitative studies, we extracted the reported association in a standardized form (prioritizing adjusted estimates when available) and recorded score handling; for qualitative and mixed-methods studies, we extracted the authors’ explicit claims regarding how digital capabilities influenced understanding or engagement. We then constructed the evidence map (Figure 3) to display evidence concentrations across populations and outcome domains.

## 3 Results

### 3.1 Study selection

The study selection process is summarized in Figure 1, reported in accordance with PRISMA-ScR guidance. Searches identified 217 records in total, including 97 from PubMed and 120 from Scopus. After removing 47 duplicate records, 170 records remained for title and abstract screening. Of these, 101 records were excluded at the title and abstract stage because they did not meet eligibility criteria, most commonly due to an ineligible population (not cancer patients, survivors, or oncology health workers), absence of an explicit eHealth literacy or digital health literacy measure (reporting digital behavior only), or non-primary publication type (for example, reviews, protocols, or abstracts without full results).

We sought full texts for 68 reports. Three reports could not be retrieved or contained insufficient abstract information to support full eligibility assessment. The remaining 65 reports were assessed in full text. No full-text exclusions occurred (n = 0), and 65 studies were included in the scoping review.

### 3.2 Overview of included studies

**Figure 4:**
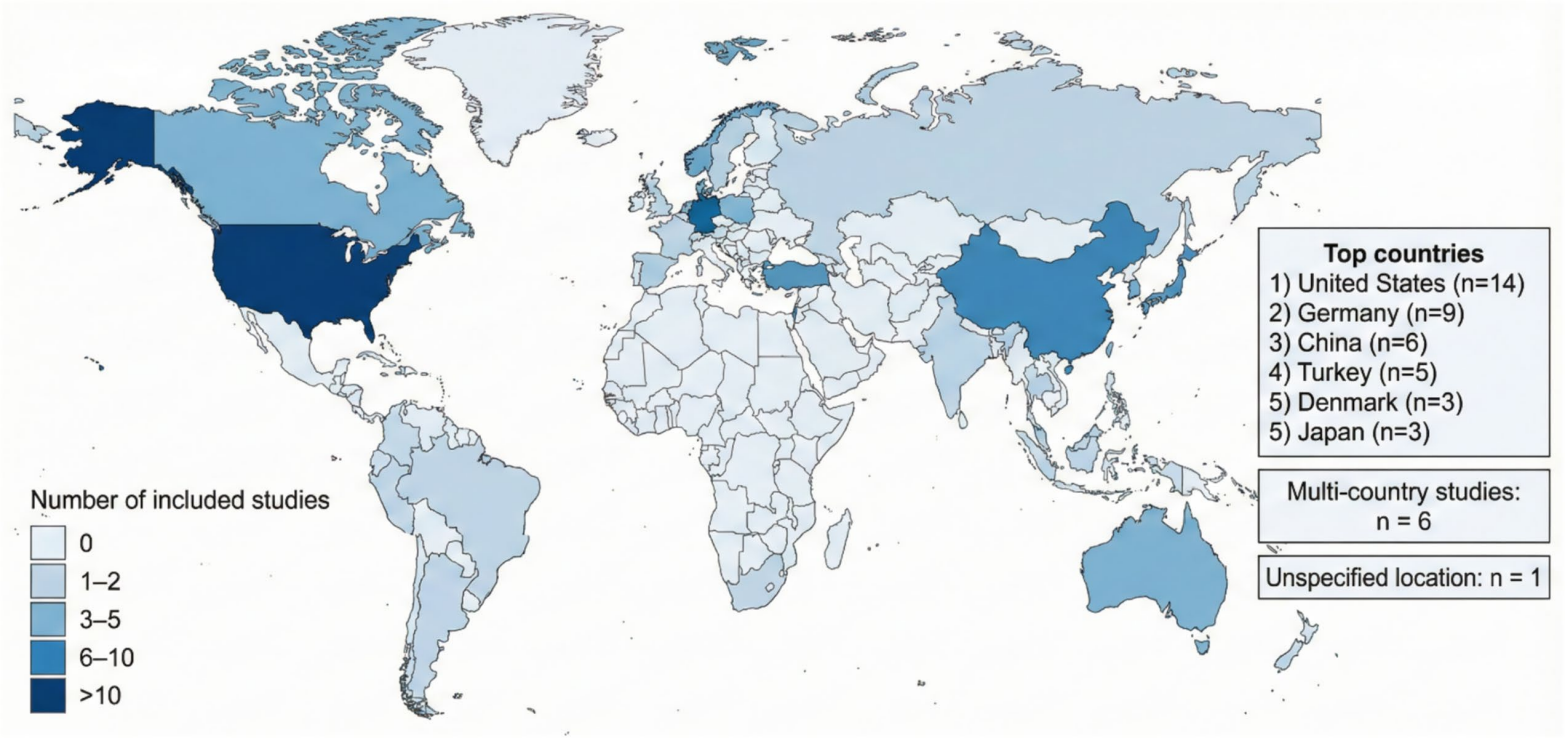
Geographic distribution of the included primary studies.

Study-level characteristics for all included studies are provided in Supplementary Dataset S1 (extracted study-level dataset), including publication year, country or region of data collection, study design, population type, cancer type, sample size, the eHealth literacy or digital health literacy instrument used, and the outcome domain(s) assessed. The geographic distribution of included evidence is shown in Figure 4.

### 3.3 Populations and oncology contexts

Across the included evidence base, study populations were predominantly patient-facing, with smaller bodies of work focused on survivorship and oncology health workers. In the same dataset, 6 studies (9.7%) focused primarily on cancer survivors, and 4 studies (6.5%) examined health workers in oncology-related roles. In total, 31 studies (50.0%) focused on cancer patients, 6 studies (9.7%) on cancer survivors, and 4 studies (6.5%) on oncology health workers.

Several studies used mixed samples, most commonly combining patients with caregivers or family members (5 studies, 8.1%) or combining patients and survivors (3 studies, 4.8%). A small number of studies targeted screening-eligible participants (1 study, 1.6%) or broader participant groups described as general or mixed populations (8 studies, 12.9%), with 3 studies (4.8%) coded as other or unclear due to non-standard population labels. The cross-classification of population group by cancer type is provided in Table 1 (Population-by-cancer matrix).

Cancer contexts were frequently not site-restricted. The most common cancer framing was mixed or “any cancer” cohorts (23 studies, 37.1%), reflecting samples drawn from heterogeneous oncology populations or survivorship programs. Among site-specific studies, breast cancer was most frequently represented (14 studies, 22.6%), followed by cervical cancer (6 studies, 9.7%). Smaller subsets focused on lung cancer (3 studies, 4.8%), prostate cancer (3 studies, 4.8%), and colorectal cancer (2 studies, 3.2%). A small number addressed other site-specific contexts (2 studies, 3.2%) or less common groups such as bladder cancer, skin/melanoma, and pediatric cancers (each 1 study, 1.6%).

### 3.4 eHealth literacy measurement and score handling

The included literature used a diverse set of eHealth literacy and digital health literacy measures, with substantial concentration around a small number of instruments. Table 2 provides a structured summary of instruments and analytic handling.

**Table 2:** Summary of eHealth literacy instruments and analytic scoring methods.

Measurement tool (group)	Studies (n)	Percent (%)
(A) eHealth literacy / digital health literacy measurement tool		
eHEALS family	41	66.1
DHLI	0	0
eHLQ/eHLA/READHY	4	6.5
Other / study-specific	16	25.8
Not reported/unclear	1	1.6
(B) eHL score handling in analysis		
Continuous	21	33.9
Categorized/threshold-based	6	9.7
Both continuous and categorized	21	33.9
Not applicable/qualitative-only	11	17.7
Not reported/unclear	3	4.8

Denominator: *N* = 65 unique studies in the extraction dataset. Percentages are out of N.

Among studies with extractable measurement details in the current dataset (*n* = 65 studies with fully charted tool fields), the eHEALS family was the dominant approach (44/65, 70.0%). Use of more skills-oriented or multidimensional instruments was comparatively limited. Only 1/65 studies (1.5%) used the Digital Health Literacy Instrument (DHLI), and 4/65 studies (6.4%) used broader frameworks such as eHLQ/eHLA/READHY. A small subset applied other or study-specific measures (3/65, 4.8%), often adapting eHEALS-like items to match a specific platform or content context. Finally, 10/65 studies (16.0%) had tool labels recorded as not specified or unclear in the extracted fields, reflecting incomplete reporting within the extraction file and signaling where instrument identification may need reconfirmation at full text.

Analytic handling of eHealth literacy scores also varied. The most common approach treated eHL as a continuous variable (21/65, 33.7%), followed by categorized or threshold-based handling (15/65, 24.0%), and models using both continuous and categorized representations (12/65, 19.4%). A smaller segment of records in the extracted dataset indicated no quantitative score or unclear score handling (14/65 combined, 22.6%), which will be reflected transparently in the mapping and interpreted cautiously in later synthesis.

### 3.5 Outcomes and measures linked to eHealth literacy

Across the extracted dataset (65 unique included studies currently charted), outcomes linked to eHealth literacy and digital health literacy were heterogeneous in both content and measurement, and many studies reported more than one eligible outcome. At the synthesis stage, we therefore treated each distinct eHL to outcome relationship as a separate *study-outcome linkage* for evidence mapping. In total, we charted 77 study-outcome linkages across the 65 studies.

Most linkages clustered into a small number of outcome domains aligned with cancer-related “insight” and engagement. The most frequently represented domain was cancer knowledge and awareness (including risk knowledge, attitudes, and awareness-related constructs), accounting for 22/77 linkages (28.6%) and appearing in 20 studies. The second largest cluster reflected digital service use and readiness outcomes (for example, intention to use digital health services, uptake of digital support tools, acceptability, usability, readiness, or implementation-related outcomes), representing 15/77 linkages (19.5%) across 13 studies. A third major cluster focused on information appraisal and misinformation-related competencies, including trust, credibility judgments, information-seeking and information-quality constructs, and misinformation susceptibility where reported (12/77 linkages, 15.6%; 12 studies). Screening behavior or screening intention was also prominent (9/77 linkages, 11.7%; 8 studies), capturing outcomes such as participation, intention, follow-up behaviors, or prevention-related behaviors within cancer screening contexts.

Less frequently, studies linked eHealth literacy to treatment adherence or self-management outcomes (3/77 linkages, 3.9%; 3 studies) and to decision-making constructs (3/77 linkages, 3.9%; 3 studies), such as preference-sensitive decisions or communication/choice-related outcomes. A small subset assessed broader patient-reported outcomes including quality of life (3/77 linkages, 3.9%; 3 studies) and psychological outcomes (2/77 linkages, 2.6%; 2 studies). The remaining outcomes (8/77 linkages, 10.4%) were labeled inconsistently across studies and were retained for mapping but interpreted cautiously due to limited standardization.

Outcome measurement approaches varied substantially. Many studies used study-specific questionnaires or purpose-built Likert items tailored to the cancer context. A smaller subset used clearly identifiable, validated instruments for broader outcomes (e.g., established quality-of-life or toxicity-related scales), but these were not the dominant pattern in the current evidence base. To make this heterogeneity transparent and usable for synthesis, we provide Supplementary Table S2 (Outcomes and measures crosswalk), which links each outcome domain to the specific outcome measures and metrics used across studies, distinguishing standardized instruments from study-developed measures where possible.

### 3.6 Evidence map of eHealth literacy to outcome associations

To synthesize a heterogeneous evidence base without forcing premature meta-analysis, we constructed an evidence map that treats each included study as contributing one or more study-to-outcome-domain linkages (for example, a single study could link eHealth literacy to both screening intention and cancer knowledge). Figure 3 visualizes where the evidence concentrates across population groups (patients, survivors, oncology health workers, mixed samples, and general or screening populations) and outcome domains. The direction of associations is communicated directly in the evidence map using the color coding described in the Figure 3 legend (positive, negative, null, mixed/depends, or unclear), while cell values represent the number of studies to outcome-domain linkages.

Across the 65 unique studies currently charted in the extraction dataset, the evidence map contained 75 study-to-domain linkages, reflecting multiple outcomes were frequently reported within the same study. Evidence clustered most strongly in domains related to patients’ digital engagement and information processing. The highest-density domains were digital service use and readiness (16 studies contributing at least one linkage), information appraisal and misinformation-related constructs (15 studies), and knowledge and awareness (14 studies). By contrast, fewer studies linked eHealth literacy to downstream clinical or well-being outcomes, including screening behavior or intention (8 studies), decision-making outcomes (4 studies), adherence or self-management (3 studies), and quality of life (3 studies). Psychological outcomes were rarely charted as a primary linkage in the current dataset (1 study).

The distribution across populations showed a strong patient-centered focus. Patients accounted for the majority of mapped linkages (39 of the 75 study-to-domain linkages), spanning all major outcome domains. The densest patient cells in the map were patients by digital service use/readiness (10 studies), patients by information appraisal/misinformation (7 studies), and patients by knowledge/awareness (6 studies). Mixed samples that included patients and caregivers also contributed meaningfully (11 linkages), particularly in digital service use/readiness (3 studies), information appraisal/misinformation (3 studies), and knowledge/awareness (2 studies). Evidence among survivors was comparatively sparse (6 linkages total, typically single-study contributions across several domains), while oncology health workers were represented in only a small number of mapped linkages (1 linkage in the current dataset), indicating a clear gap in workforce-focused research linking eHealth literacy to oncology-related practice or patient-facing outcomes. Taken together, the evidence maps highlight two key features of the field that guide subsequent synthesis. First, studies most commonly operationalize the “insight” component through knowledge, awareness, and information appraisal, and through proximal markers of engagement with digital services. Second, there is comparatively limited mapped evidence connecting eHealth literacy to more distal outcomes such as adherence, quality of life, and psychological outcomes, and very limited work in oncology health worker populations. These patterns, and the reported directionality of associations summarized in Figure 3, inform the interpretation of where evidence is robust versus where important gaps remain.

### 3.7 Heterogeneity assessment and implications

The included studies exhibited substantial heterogeneity across multiple dimensions. Clinical heterogeneity was evident in the diversity of cancer types, disease stages, and population groups (patients, survivors, and health workers). Methodological heterogeneity arose from variation in study designs, with most studies being cross-sectional and a minority employing longitudinal or mixed-methods approaches. Measurement heterogeneity was also prominent, with wide variation in eHealth literacy instruments, outcome definitions, and analytic strategies.

This heterogeneity limits direct comparability across studies and precludes quantitative synthesis. It also suggests that observed associations may be context-dependent, influenced by population characteristics, measurement tools, and healthcare settings.

### 3.8 Summary of principal findings

This scoping review mapped how electronic health literacy (eHealth literacy) and closely related constructs (digital health literacy) have been studied in oncology-relevant populations, and how these literacy measures have been linked to “insight-like” outcomes such as cancer knowledge, screening behaviors, information appraisal, and downstream psychosocial or quality-of-life endpoints.

Across the included evidence base, research attention clustered in patient-facing digital engagement and information-processing outcomes, with thinner coverage for outcomes closer to clinical endpoints. In the extraction dataset available for synthesis here, the most frequently mapped outcome domains were digital service use/readiness (16 studies), information appraisal and misinformation-related constructs (15 studies), and knowledge/awareness (14 studies). By contrast, fewer studies linked eHealth literacy to screening behavior/intention (8 studies), decision-making outcomes (4 studies), adherence/self-management (3 studies), quality of life (3 studies), or explicitly charted psychological outcomes (2 studies). Notably, no studies were mapped to symptom awareness outcomes based on the extracted outcome labels, leaving an important “early recognition and help-seeking” pathway largely unexamined in this evidence base.

The distribution of evidence across populations suggests that the field is still predominantly patient-centered, with limited attention to the oncology workforce and survivorship as distinct contexts. In the same synthesis dataset, patients accounted for 31 studies, and mixed or other samples (for example, patient-caregiver or blended cohorts) accounted for 18 studies. Survivors represented 9 studies, while oncology health workers were represented in only 3 studies, and explicitly screening-eligible populations in 1 study. This imbalance matters because the practical implications of eHealth literacy differ by role and phase of care. Patients use digital information to interpret diagnoses and navigate services, survivors often manage long-term monitoring and late effects, and health workers increasingly mediate digital care pathways and patient-facing risk communication.

A second principal finding concerns measurement choice. Most studies relied on perceived-skill measures, especially eHEALS or close variants, which were used in 41 of 65 studies (66.0%) in the charted dataset. eHEALS is widely used because it is short and easy to administer, capturing perceived comfort and skills in finding, evaluating, and applying online health information. However, the field shows limited uptake of more comprehensive tools designed to reflect multiple dimensions of digital health engagement. Multidimensional instruments such as eHLQ were rarely used (4 of 65 studies), and there were no studies using DHLI in the charted dataset, despite its focus on navigation, reliability appraisal, and privacy-related competencies. This pattern suggests a measurement gap: the literature often quantifies “how confident people feel” rather than also testing “what people can do” in digital environments shaped by platform design and misinformation dynamics.

Third, we observed substantial heterogeneity in analytic handling of eHealth literacy scores, which complicates cross-study comparison even when the same tool is used. Within the extraction dataset, studies frequently modeled eHealth literacy as continuous (23 of 65) and reported both continuous and categorized approaches (22 of 65). A smaller set used purely threshold-based categories (6 of 65), while qualitative studies (11 of 65) treated eHealth literacy-related constructs descriptively, and a minority did not clearly report handling (3 of 65). Because cut-offs and grouping strategies can alter apparent associations, inconsistent score handling can produce results that look incompatible even when underlying patterns are similar.

Finally, the mapped evidence highlights a consistent conceptual theme: in oncology contexts, “insight” has most often been operationalized through knowledge, awareness, and information appraisal behaviors, rather than through clinically proximal behaviors (adherence) or patient-centered endpoints (quality of life, distress). This reflects a plausible pathway in which eHealth literacy shapes a person’s ability to search, evaluate, and integrate cancer information, which then influences decisions and actions. Still, the current distribution of outcomes suggests that future work needs to test whether these proximal informational gains translate into durable behavioral change and improved well-being, particularly in populations most at risk of digital exclusion.

Taken together, these findings indicate that the literature has developed a strong base for understanding eHealth literacy in relation to digital engagement and informational outcomes in cancer populations, but it remains underdeveloped for (1) workforce and survivorship contexts, (2) symptom awareness and help-seeking pathways, (3) adherence and quality-of-life outcomes, and (4) measurement approaches that assess multidimensional and performance-based digital competencies. These concentrations and gaps provide the foundation for the targeted priorities summarized later in Table 2, and they frame interpretation across outcome domains.

### 3.9 Interpretation of the evidence in relation to existing literature

The pattern observed in our evidence map, with the strongest concentration of studies linking eHealth literacy to digital service use/readiness, information appraisal or misinformation-related constructs, and knowledge/awareness, is consistent with how eHealth literacy has historically been conceptualized and measured. Early foundational work positioned eHealth literacy as a combined set of perceived skills and comfort in finding, evaluating, and applying online health information. This framing naturally aligns with outcomes that sit close to information seeking and digital navigation, rather than with distal clinical or well-being endpoints.

At the same time, our findings underscore a persistent measurement and comparability issue in this field. Most included studies relied on brief self-report scales, especially eHEALS, and far fewer used multidimensional instruments or performance-based components.

While widely used, eHEALS captures perceived confidence rather than objective performance, which may lead to overestimation of actual digital competencies. In contrast, instruments such as the Digital Health Literacy Instrument (DHLI) incorporate performance-based elements, and multidimensional tools such as the eHealth Literacy Questionnaire (eHLQ) capture broader interactions between individuals and digital health systems. The limited use of these instruments in the included studies restricts insight into functional and context-dependent digital skills.

The strong emphasis on information appraisal and misinformation-related outcomes in our synthesis is also consistent with the rapidly evolving cancer information ecosystem. Cancer-related information is increasingly encountered through “new media” channels (social platforms, video platforms, and emerging AI-mediated sources), where content quality is uneven and where persuasive but inaccurate claims can spread quickly. This context strengthens the plausibility of a pathway in which eHealth literacy operates as a protective factor by improving appraisal skills and supporting calibrated trust. However, the mapped evidence in our dataset still links eHealth literacy more often to appraisal constructs and engagement behaviors than to downstream outcomes such as adherence, quality of life, or psychological distress.

Our identified gaps can also be interpreted as a consequence of outcome selection and study design choices. Many studies were cross-sectional and prioritized outcomes that are relatively easy to collect via single-time surveys. In contrast, outcomes like adherence, quality of life, or symptom recognition and help-seeking typically require longitudinal follow-up, linkage to clinical records, or validated patient-reported outcome instruments, and they are more sensitive to confounding by disease stage, treatment burden, and socioeconomic factors.

Finally, the limited representation of oncology health workers and the small number of survivorship-focused studies suggest that current evidence is still oriented toward the “patient as information consumer” model. In real oncology settings, eHealth literacy is shaped by system design, clinician communication practices, and the availability of trustworthy digital pathways. A more mature evidence base would therefore connect eHealth literacy not only to what patients know or click, but also to how digital competencies interact with care delivery, including workforce digital practices and survivorship self-management demands.

### 3.10 Implications for practice, policy, and future research

The evidence mapped in this review indicates that eHealth literacy is increasingly recognized as shaping how people affected by cancer use digital information and services. However, it is not yet consistently embedded in clinical workflows, service design, or intervention research. Based on the main concentrations and gaps (Supplementary Table S4), we highlight three practical implications.

First, oncology services should treat eHealth literacy as a practical “fit” between people and digital systems, not as a fixed personal trait. This supports a tiered model of digital support: (1) universally accessible portals and information that follow plain-language and usability principles, (2) targeted help for patients more likely to face digital barriers (for example, older age, lower education, or limited access), and (3) intensive navigation support for those who continue to struggle. This approach reflects persistent disparities in portal access and sustained use in oncology, which can limit equitable participation in care processes such as viewing results, messaging clinicians, and completing patient-reported outcomes.

Second, the concentration of evidence around information appraisal and digital readiness points to a priority: strengthening resilience to misinformation within cancer communication. Cancer misinformation can delay evidence-based care, promote harmful practices, and weaken trust in professional advice. Practical steps include clinician-endorsed lists of trustworthy resources, brief coaching on credibility cues (such as source transparency, conflicts of interest, and the quality of the underlying evidence), and structured opportunities for patients to bring online information into consultations without discomfort. In parallel, cancer centers and professional societies can improve the broader information environment by producing shareable, high-quality content and making it easier to find.

Third, improving outcomes will likely require interventions that address both patients and the oncology workforce, because digital care is co-produced. Portals, remote monitoring, and digital decision aids depend on staff capacity to onboard patients, troubleshoot barriers, and communicate effectively through digital channels. Evidence on oncology health workers was limited in this review, supporting a system-level priority of structured digital skills training for oncology professionals, including staff involved in patient support, aligned with real tasks such as portal workflows, digital communication, and safe use of emerging tools.

These implications also shape the research agenda. The field remains dominated by perceived-skill measures, with fewer studies using multidimensional or competence-oriented instruments. Future work should more often include skill-anchored measurement, especially for misinformation handling and navigation. Finally, the field would benefit from more longitudinal and intervention designs that test whether improving digital competencies, or improving service usability, leads to meaningful downstream outcomes for patients, including adherence-related behaviors, distress, and quality of life.

Overall, digital inequities in cancer care are rarely explained by skills alone. Access constraints, usability barriers, and workflow limitations can magnify the impact of low eHealth literacy. The most defensible path forward is an integrated approach that measures and supports patient capability while improving digital service design and workforce readiness.

### 3.11 Equity and digital divide considerations (age, education, access, context moderators as extracted)

Equity is central to interpreting eHealth literacy in oncology because observed associations can reflect both individual capability and structural opportunity. In this review, equity-relevant synthesis was limited by incomplete reporting and incomplete extraction fields. Age was missing in a majority of studies (mean age reported in 24/65), which restricts interpretation because age is a plausible modifier of operational skills, navigation confidence, and uptake of digital services. Sex composition was also missing in a subset of studies, and many samples were female-skewed, partly reflecting cancer-site focus and partly reflecting recruitment routes that may overrepresent digitally connected groups.

Education, socioeconomic resources, and digital access are likely key moderators, but these variables were not consistently available in the structured dataset used for synthesis. This is a major limitation because eHealth literacy and digital opportunity are intertwined; without these fields, equity-aware comparisons remain tentative. Geographic context further limits generalizability because the included evidence clustered in high-income settings, where the demands and availability of portals and digital services differ from resource-limited contexts.

Future oncology eHealth literacy studies should treat core equity variables as minimum reporting items (age, education, access proxies, recruitment source, and missingness) and test effect modification when sample size allows. In parallel, interventions should combine capability support with usability-driven service design and non-digital alternatives to avoid widening disparities.

### 3.12 Strengths and limitations of the evidence base and this review

A central strength of this review is the use of a transparent scoping approach to map a heterogeneous literature, rather than forcing premature quantitative synthesis. We followed an established scoping framework and reported search, selection, and charting in line with PRISMA-ScR expectations. A second strength is the structured separation of study populations, how eHealth literacy was operationalized, and which cancer-related outcomes were assessed.

In line with established scoping review methodology, we did not perform a formal risk-of-bias assessment of included studies. However, we systematically examined key methodological characteristics to inform interpretation. Most included studies were cross-sectional and relied on self-reported measures, which limits causal inference and introduces potential reporting bias. The predominance of perceived-skill instruments (e.g., eHEALS) further constrains interpretation, as these tools may reflect confidence rather than actual performance in digital environments.

Overall, the literature supports a structured evidence map and gap identification, but remains too inconsistent in design, measurement, and outcomes to support strong causal conclusions or detailed comparative claims across cancers and contexts.

To guide interpretation, we use a mechanism-oriented pathway: eHealth literacy influences (1) entry into digital services and information sources, (2) information processing skills such as navigation, credibility appraisal, and relevance determination, and (3) proximal outcomes such as knowledge, appraisal behaviors, and engagement, which may translate into distal outcomes including screening follow-up, adherence/self-management, and quality of life. These relationships are likely moderated by sociodemographic resources, access and usability of digital services, clinical context (type, stage, treatment burden), and the surrounding information environment, including misinformation prevalence. This framework clarifies why the current evidence clusters around knowledge, appraisal, and readiness, while links to symptom awareness, adherence, and patient-centered outcomes remain under-tested.

In summary, this framework clarifies why the current evidence base clusters around knowledge, appraisal, and digital readiness, while highlighting largely untested links to symptom awareness, adherence, and patient-centered outcomes. It provides a structured, mechanism-informed, and equity-aware basis for interpreting the evidence map and for designing the next generation of oncology eHealth literacy studies and interventions.

## 4 Conclusion

This scoping review mapped 65 primary studies linking eHL or related digital health literacy constructs to cancer-relevant outcomes across patients, survivors, and oncology health workers, using a PRISMA-ScR reporting approach. The evidence base is most mature for outcomes close to digital engagement and information processing, including digital service use/readiness and information appraisal or misinformation-related constructs. In contrast, there is comparatively limited and fragmented evidence linking eHL to downstream outcomes such as adherence/self-management, psychological outcomes, and quality of life, and our mapped dataset showed no explicit coverage of symptom-awareness outcomes, despite their relevance to timely help-seeking.

Two practical conclusions follow. First, the field largely measures eHL using brief self-report tools (most commonly eHEALS), which capture perceived competence but may not fully reflect real-world performance in navigation, credibility appraisal, and privacy behaviors. Second, the current dominance of cross-sectional designs and heterogeneity in score handling limit causal interpretation and inhibit comparisons across settings and populations. As a result, the most defensible interpretation at present is that eHL is plausibly linked to key “insight-adjacent” mechanisms (knowledge, appraisal, engagement), but stronger study designs are needed to confirm pathways to patient-centered outcomes. In summary, the next phase of this research field should shift from documenting that “eHL matters” to specifying what to change, for whom, and in which care contexts, using stronger measures, equity-aware designs, and outcomes that reflect the realities of modern cancer care.
