# Supplementary material for: Role of electronic health literacy and insight into cancer among health workers and patients: a review
Source: Front Public Health. 2026 May 29;14:1781759. doi: 10.3389/fpubh.2026.1781759 (PMC13260071; doi:10.3389/fpubh.2026.1781759)
Supplement: Supplementary file 1 [file Supplementary_file_1.DOCX]

Supplementary materials

*Table S1. Eligibility criteria defined by the PCC (Population, Concept, Context) framework.*

| Domain | Include | Exclude | Decision notes |
| --- | --- | --- | --- |
| Population | Cancer patients (any type/stage/phase), cancer survivors, or oncology health workers. General population only if explicitly focused on cancer screening/awareness. | Non-cancer populations; non-oncology health workers; caregiver-only samples; general public without cancer screening/awareness focus. | Cancer must be central (not background-only). |
| Concept: eHealth literacy | Explicit measurement of eHealth literacy / digital health literacy using validated tools (eHEALS, DHLI, eHLQ-related, eHLA/READHY) or clearly defined construct-consistent measures. | General health literacy only; internet/portal/social media use without literacy/skills measurement. | Exposure must be literacy/skills, not just technology use. |
| Outcomes linked to eHL | eHL linked to ≥1 cancer-relevant outcome: knowledge/awareness, symptom awareness, screening participation/intention, adherence/self-management, misinformation appraisal/info quality, decision-making outcomes, or psychological outcomes (anxiety, empowerment, self-efficacy, distress). | eHL measured but not linked to outcomes; outcomes unrelated to these domains. | “Link” can be quantitative association or qualitative thematic linkage. |
| Study type | Primary empirical studies (quantitative, qualitative, mixed methods, trials). | Reviews/meta-analyses; editorials/commentaries/letters; protocols; conference abstracts without full results. | Must report original data and results. |
| Context | Any setting/country. | Cancer context peripheral only. | Broad by design. |
| Language / time | English; no date limits. | Non-English. | Full text must be in English. |
| Availability / duplicates | Full text available; keep most complete report per study. | Abstract-only; duplicate or superseded reports. | If same dataset appears in multiple papers with distinct eligible outcomes, retain both and note overlap during extraction. |

*Table S2. Crosswalk of outcome domains linked to specific measurement instruments.*

| Outcome domain | n studies | % of studies | Common outcome labels (top) | Common measures/metrics (top) |
| --- | --- | --- | --- | --- |
| Other / mixed outcomes | 19 | 30.6 | Cancer prevention behavior (use of digital technologies for cancer prevention) and broader digital health behaviors (n=1); Digital health engagement, health equity and disparities in vulnerable populations (n=1); Digital health literacy in relation to treatment satisfaction, preferences and health outcomes (non-cancer) (n=1); Implementation and digital skills/digital health literacy as barriers or facilitators of ePRO use in cancer care (n=1); Online informational and emotional support-seeking behavior (n=1); Patient experience, communication, and access to genetic counseling (including the role of digital health literacy and language proficiency) (n=1); … (+2 more) | eHEALS total score (n=2); EORTC QLQ-C30 quality-of-life domains (n=1); emergency department visits and hospitalizations (n=1); adherence to scheduled cystoscopy follow-up (n=1); Intention to Use Digital Healthcare Services scale (adapted from technology acceptance / AI use intention items) (n=1); … (+5 more) |
| Knowledge/awareness | 14 | 22.6 | cancer knowledge/awareness (n=2); Cancer education and navigation training outcomes (including eHealth literacy) (n=1); Cancer knowledge/awareness (cancer health literacy status) (n=1); Cancer knowledge/awareness (colorectal cancer knowledge gain after website exposure) (n=1); Cancer knowledge/awareness and digital health literacy (n=1); Cancer knowledge/awareness and online information seeking behavior (n=1); … (+2 more) | composite reproductive health literacy and knowledge indices (n=1); HLS-EU-Q6 general HL score (n=1); eHEALS digital HL score (n=1); qualitative themes on media portrayal and medical mistrust (n=1); HPV vaccine belief items (n=1); … (+5 more) |
| Information appraisal/misinformation | 13 | 21 | App acceptability, cultural sensitivity and information needs in survivorship (n=1); Cancer fatalism beliefs in relation to online cancer information seeking (n=1); Cancer information needs, information-seeking behavior, and digital health literacy challenges (n=1); Digital engagement, health information seeking, and health equity (including cancer-related contexts) (n=1); Digital information needs, experiences using online health information and patient portals, and perceived connection to care (n=1); Health literacy environment / readability and quality of online cancer information (n=1); … (+2 more) | Self-developed items on utilization and attitudes towards digital support tools and AI (n=1); binary indicators of use vs. non-use of digital support tools (n=1); trust in new technologies rated 0–10 (n=1); Latent classes of online health information seeking (eg, information explorers, occasional seekers, information experts) based on frequency, channels and topics of online information seeking. (n=1); Flesch-Kincaid Grade Level, Gunning Fog Index, Simple Measure of Gobbledygook (SMOG), and DISCERN Quality Index (n=1); … (+5 more) |
| Screening behavior/intention | 8 | 12.9 | Cancer knowledge/awareness (CRC) and screening behavior (n=1); Cancer prevention intervention evidence (apps for behavior change and screening) (n=1); Cancer screening attitudes / intention to participate in screening (n=1); Cancer screening behavior (Pap smear/HPV DNA test participation), online health information seeking and cyberchondria (health anxiety related to online searching) (n=1); Cancer screening intention and health information behaviours (social media use, trust in cancer information) (n=1); HPV vaccination uptake (n=1); … (+2 more) | Attitude Scale for Cancer Screening (ASCS) total score (n=1); qualitative themes on media portrayal and medical mistrust (n=1); Cervical cancer knowledge items based on CDC fact sheet (n=1); self-reported Pap smear receipt and HPV vaccination status (n=1); Pap Smear Belief Questionnaire (n=1); … (+5 more) |
| Digital service use/readiness | 7 | 11.3 | Digital engagement / patient portal use (n=1); Digital skills, training needs, and readiness for digital health in cancer care (n=1); Health technology readiness / eHealth engagement profile (n=1); Usability, user experience and acceptability of a cancer prevention mobile app (n=1); app usability and acceptability (n=1); eHealth / technology readiness and enablement (n=1); … (+1 more) | Self-rated performance and perceived importance of specific digital skills (for example, using patient portals, telehealth tools, electronic records) and composite gap indicators from importance–performance analysis. (n=1); likelihood of future app use (n=1); eHEALS digital health literacy score used to stratify analyses. (n=1); perceived difficulty and confidence ratings for 10 predefined tasks (n=1); Task success rate and completion time (n=1); … (+5 more) |
| Decision making | 4 | 6.5 | Decision making outcomes (decisional confidence, trust/credibility perceptions) (n=1); Financial toxicity & decision-making preference (n=1); Internet and eHealth service usage, perceived usefulness for decision making, and confidence in handling online health information (n=1); Treatment decision making involvement and information seeking (n=1) | Comprehensive Score for financial Toxicity (COST) and Control Preference Scale (CPS) for financial decision-making (n=1); Partner involvement in eight treatment-decision activities (yes/no) and size of social support network scale (0–14) (n=1); Frequency of eHealth service use, item ratings on understanding, finding and using online information, perceived usefulness for decisions, and Single Item Literacy Screener (n=1); Perceived trustworthiness/credibility and decisional confidence measures (names not specified in provided file) (n=1) |
| Adherence/self-management | 3 | 4.8 | Conceptual discussion of survivorship care, self-management and (digital) health literacy (n=1); medication adherence and ADR management (n=1); treatment adherence (cystoscopy follow-up) (n=1) | EORTC QLQ-C30 quality-of-life domains (n=1); emergency department visits and hospitalizations (n=1); adherence to scheduled cystoscopy follow-up (n=1); MMAS-8 (Morisky Medication Adherence Scale) embedded in the app for adherence scoring (n=1); Qualitative themes on usability and utility (n=1); … (+2 more) |
| Quality of life | 3 | 4.8 | eHealth literacy level (self-perceived) and quality of life correlates (n=1); health-related quality of life (n=1); quality of life (n=1) | EORTC QLQ-C30 quality-of-life domains (n=1); emergency department visits and hospitalizations (n=1); adherence to scheduled cystoscopy follow-up (n=1); HLQ domains (especially ‘understanding health information enough to know what to do’), eHLQ domains, and RAND-12 physical and mental component scores (n=1); eHEALS (n=1); … (+1 more) |
| Psychological outcomes | 2 | 3.2 | Psychological distress, information avoidance and compliance with COVID-19 preventive measures (n=1); caregiver burden, psychological distress, and self-efficacy (n=1) | Zarit Burden Interview (short form), Distress Thermometer, General Self-Efficacy Scale plus health literacy scales (HLCS-C, CHLT-6) (n=1); Self-reported distress from COVID-19 information, information avoidance scale, and self-reported adherence to preventive measures (n=1) |

Table S3. Summary of reported associations between eHealth literacy and cancer-related outcomes.

| Outcome domain | n (% of studies) | Top outcome labels | Top measures/metrics | Typical eHL tool(s) | Typical score handling |
| --- | --- | --- | --- | --- | --- |
| Other / mixed outcomes | 18 (29.0%) | Cancer prevention behavior (use of digital technologies for cancer prevention) and broader digital health behaviors (n=1); Digital health engagement, health equity and disparities in vulnerable populations (n=1); Digital health literacy in relation to treatment satisfaction, preferences and health outcomes (non-cancer) (n=1); …(+3) | eHEALS total score (n=2); adherence to scheduled cystoscopy follow-up (n=1); emergency department visits and hospitalizations (n=1); …(+3) | eHEALS (eHealth Literacy Scale) (n=3); Custom digital health literacy skills scale for using Kanker.nl (information-seeking, assessing, application and navigation skills; …(+4) | Both continuous (mean scores) and categorized into low, moderate, and good EHL; proportional odds models used to examine associations with demographics (n=1); …(+4) |
| Knowledge/awareness | 14 (22.6%) | cancer knowledge/awareness (n=2); Cancer education and navigation training outcomes (including eHealth literacy) (n=1); Cancer knowledge/awareness (cancer health literacy status) (n=1); …(+3) | eHEALS digital HL score (n=1); composite reproductive health literacy and knowledge indices (n=1); HLS-EU-Q6 general HL score (n=1); …(+3) | eHEALS (eHealth Literacy Scale) (n=3); GR-eHEALS (German version of eHEALS) (n=1); …(+1) | Continuous J-eHEALS score; participants grouped into low vs high eHL based on pre-survey scores (n=1); …(+4) |
| Information appraisal/misinformation | 13 (21.0%) | App acceptability, cultural sensitivity and information needs in survivorship (n=1); Cancer fatalism beliefs in relation to online cancer information seeking (n=1); Cancer information needs, information-seeking behavior, and digital health literacy challenges (n=1); …(+3) | binary indicators of use vs. non-use of digital support tools (n=1); Self-developed items on utilization and attitudes towards digital support tools and AI (n=1); trust in new technologies rated 0–10 (n=1); …(+3) | eHEALS (eHealth Literacy Scale) (n=3); No formal eHL tool; …(+3) | Categorized (above-average vs low eHealth literacy) (n=1); Continuous eHEALS score and categorical DHL levels (low/medium/high) used as predictors in logistic regression and ANOVA (n=1); …(+1) |
| Digital service use/readiness | 8 (12.9%) | Digital engagement / patient portal use (n=1); Digital skills, training needs, and readiness for digital health in cancer care (n=1); Health technology readiness / eHealth engagement profile (n=1); …(+3) | Intention to Use Digital Healthcare Services scale (adapted from technology acceptance / AI use intention items) (n=1); Self-rated performance and perceived importance of specific digital skills (for example, using patient portals, telehealth tools, electronic records) and composite gap indicators from importance–performance analysis. (n=1); likelihood of future app use (n=1); …(+3) | eHEALS (eHealth Literacy Scale) (n=2); Ad hoc online questionnaire on digital skills and training needs based on DigComp 2.2; …(+2) | Categorical threshold (eHEALS ≥ 50%) used as an inclusion criterion; scores otherwise described descriptively. (n=1); …(+4) |
| Screening behavior/intention | 8 (12.9%) | Cancer knowledge/awareness (CRC) and screening behavior (n=1); Cancer prevention intervention evidence (apps for behavior change and screening) (n=1); Cancer screening attitudes / intention to participate in screening (n=1); …(+3) | Attitude Scale for Cancer Screening (ASCS) total score (n=1); Pap Smear Belief Questionnaire (n=1); HPV vaccine belief items (n=1); …(+3) | Japanese version of eHEALS (J-eHEALS) (n=1); No dedicated eHealth literacy scale; …(+3) | Continuous (regression using score; OR per 1-point increase) (n=1); …(+3) |
| Decision making | 4 (6.5%) | Decision making outcomes (decisional confidence, trust/credibility perceptions) (n=1); Financial toxicity & decision-making preference (n=1); Internet and eHealth service usage, perceived usefulness for decision making, and confidence in handling online health information (n=1); …(+1) | Comprehensive Score for financial Toxicity (COST) and Control Preference Scale (CPS) for financial decision-making (n=1); Partner involvement in eight treatment-decision activities (yes/no) and size of social support network scale (0–14) (n=1); Frequency of eHealth service use, item ratings on understanding, finding and using online information, perceived usefulness for decisions, and Single Item Literacy Screener (n=1); …(+1) | Custom 8-item eHealth literacy item set based on PERQ and eHEALS concepts; plus Single Item Literacy Screener (SILS) (n=1); …(+2) | Continuous (mean eHEALS score) and dichotomized into high vs low eHealth literacy (median split) in regression models (n=1); Continuous eHEALS total score used in t tests and multiple linear regression (n=1); …(+1) |
| Adherence/self-management | 3 (4.8%) | Conceptual discussion of survivorship care, self-management and (digital) health literacy (n=1); medication adherence and ADR management (n=1); treatment adherence (cystoscopy follow-up) (n=1) | adherence to scheduled cystoscopy follow-up (n=1); emergency department visits and hospitalizations (n=1); EORTC QLQ-C30 quality-of-life domains (n=1); …(+3) | Modified eHealth Literacy Scale (eHEALS) (n=1); None (health literacy and digital health literacy discussed conceptually; …(+3) | Categorical threshold (eHEALS ≥ 50%) used as an inclusion criterion; scores otherwise described descriptively. (n=1); …(+2) |
| Quality of life | 3 (4.8%) | eHealth literacy level (self-perceived) and quality of life correlates (n=1); health-related quality of life (n=1); quality of life (n=1) | adherence to scheduled cystoscopy follow-up (n=1); emergency department visits and hospitalizations (n=1); EORTC QLQ-C30 quality-of-life domains (n=1); …(+3) | eHEALS (n=1); eHEALS (eHealth Literacy Scale; …(+2) | Categorized (high vs low eHealth literacy groups) (n=1); both continuous (scale scores in regression) and categorized (limited vs moderate vs advanced literacy groups) (n=1); …(+1) |
| Psychological outcomes | 2 (3.2%) | Psychological distress, information avoidance and compliance with COVID-19 preventive measures (n=1); caregiver burden, psychological distress, and self-efficacy (n=1) | Zarit Burden Interview (short form), Distress Thermometer, General Self-Efficacy Scale plus health literacy scales (HLCS-C, CHLT-6) (n=1); Self-reported distress from COVID-19 information, information avoidance scale, and self-reported adherence to preventive measures (n=1) | Adapted eHEALS items for COVID-19-related online information (subjective eHealth literacy) (n=1); eHealth Literacy Scale (eHEALS) (n=1) | Continuous scale and categories used as predictors in regression models (n=1); Continuous total score; …(+1) |

*Table S4. Identified evidence gaps and corresponding priorities for future research.*

| Gap (domain) | Evidence signal (from this dataset) | Priority | Next action (practical) |
| --- | --- | --- | --- |
| Eligibility and dataset integrity | Automated grouping of Study_design suggests 4/65 (6.4%) records are review/conceptual. Cancer-type field includes… | Reconcile the master included-studies list to N=65 and re-verify eligibility for any record flagged as review/conceptual or non-cancer. | Audit log: (1) match titles/DOIs to included list, (2) correct mislabeled fields, (3) remove ineligible studies; then regenerate T2–T6 and figures. |
| Oncology health workers under-represented | Only 3/65 (4.7%) studies focus on oncology health workers. In the evidence map, health-worker evidence appears only in Knowledge/awareness (n=1 cell). | Increase primary studies linking staff eHL/DHL to clinical communication quality, digital tool adoption, and patient-facing outcomes. | Multi-site surveys plus implementation studies; include role-stratified analyses (nurses, physicians, navigators) and workplace digital workflow measures. |
| Screening-eligible populations beyond screening outcomes | Only 1/65 (1.5%) studies are explicitly screening populations. Evidence map shows screening population only contributes to Screening behavior/intention (n=1). | Study eHL/DHL in screening pathways and connect it to misinformation resilience, risk perception, and follow-up completion. | Prospective designs in screening programs; measure eHL plus trust/credibility and track actual uptake and follow-up. |
| Symptom awareness outcomes missing | Symptom awareness domain has 0 studies across all population groups (0/65). | Develop and test models linking eHL to symptom recognition and appropriate help-seeking. | Design validated symptom knowledge tasks or vignettes; measure time-to-action intentions and real-world follow-up where feasible. |
| Psychological outcomes sparse and not patient-centered | Psychological outcomes appear in only 2/65 (3.1%) studies, both in Mixed/other samples; none in patient-only or survivor-only cells. | Evaluate whether eHL buffers harmful effects of online misinformation and improves empowerment and coping. | Include validated mental health instruments (anxiety/distress) alongside eHL; test mediation by information appraisal skills. |
| Adherence and self-management outcomes limited | Adherence/self-management outcomes are reported in 3/65 (4.7%) studies (patients n=1; survivors n=1; mixed n=1). | Link eHL to objective or clinically recorded adherence behaviors and self-management indicators. | Use EHR-derived follow-up attendance or pharmacy refill measures where possible; complement with self-management scales. |
| Quality of life outcomes limited | Quality of life is reported in 3/65 (4.7%) studies (patients n=2; survivors n=1). | Test pathways from eHL to QoL via service access, decision support, and self-management. | Use validated QoL instruments and include confounder adjustment (age, education, disease stage) in analytic plans. |
| Measurement tool diversity and objective assessment | eHEALS family dominates (41/65, 66.0%). No DHLI studies (0/65). Multidimensional tools (eHLQ/eHLA/READHY) are rare (4/65, 6.5%). Other/ad hoc tools are common (17/65, 25.8%). | Adopt multidimensional and performance-based digital literacy measures and report scoring transparently. | Combine self-report (eHEALS/eHLQ) with task-based assessments (credibility judgment tasks, navigation tasks) aligned to oncology contexts. |
| Study design strength (causal inference and implementation) | Broad design grouping suggests cross-sectional studies dominate (44/65, 71.0%). Trials are few (2/65, 3.1%), and longitudinal cohorts are rare (1/65, 1.6%). | Increase longitudinal and intervention studies testing whether improving eHL improves outcomes. | Embed eHL interventions into digital oncology programs; use pre–post designs with controls or pragmatic trials, and track real-world digital use. |
